# Efficacy of alcohol‐based hand sanitizers against human norovirus using RNase‐RT‐qPCR with validation by human intestinal enteroid replication

**DOI:** 10.1111/lam.13393

**Published:** 2020-10-04

**Authors:** B.I. Escudero‐Abarca, R.M. Goulter, J.W. Arbogast, R.A. Leslie, K. Green, L.‐A. Jaykus

**Affiliations:** ^1^ Department of Food Bioprocessing and Nutrition Sciences North Carolina State University Raleigh NC USA; ^2^ GOJO Industries Inc Akron OH USA

**Keywords:** disinfection, food safety, human intestinal enteroids, human norovirus, RT‐qPCR

## Abstract

Successful human norovirus (HuNoV) cultivation in stem cell‐derived human intestinal enteroids (HIE) was recently reported. The purpose of this study was to evaluate the anti‐HuNoV efficacy of two alcohol‐based commercial hand sanitizers and 60% ethanol by suspension assay using RNase‐RT‐qPCR, with subsequent validation of efficacy by HuNoV cultivation using the HIE model. In suspension, when evaluated by RNase‐RT‐qPCR, 60% ethanol resulted in less than one log_10_ reduction in HuNoV genome equivalent copies (GEC) regardless of contact time (30 or 60s) or soil load. The two commercial products outperformed 60% ethanol regardless of contact time or soil load, providing 2·2–3·2 log_10_ HuNoV GEC reductions by suspension assay. Product B could not be validated in the HIE model due to cytotoxicity. Following a 60s exposure, viral replication in the HIE model increased 1·9 ± 0·2 log_10_ HuNoV GEC for the neutralization (positive) control and increased 0·9 ± 0·2 log_10_ HuNoV GEC in challenged HIE after treatment with 60% ethanol. No HuNoV replication in HIE was observed after a 60 s exposure to Product A.

## Graphical abstract

**Significance and Impact of the Study:** The purpose of this study was to evaluate the efficacy of two alcohol‐based commercial hand sanitizers and 60% ethanol by suspension assay using RNase‐RT‐qPCR, with subsequent validation of efficacy by human norovirus (HuNoV) cultivation using stem cell‐derived human intestinal enteroids (HIE). When HuNoV was treated with hand sanitizers, a 2·2–3·2 log_10_ reduction was observed using RNase‐RTqPCR. Adsorption to HIE was inefficient, and replication was nonexistent at 72 h post infection, suggesting sanitizer treatment affects capsid binding and infectivity. Validating molecular data using an infectivity model is an important advancement towards more reliable characterization of anti‐HuNoV efficacy of sanitizers.

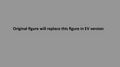

## Introduction

Human norovirus (HuNoV) is the leading cause of acute non‐bacterial gastroenteritis in industrialized countries (Glass *et al*. 2009), as well as the single most significant cause of foodborne illnesses (Scallan *et al*. 2011). It is estimated that approximately 20 million people are infected by this viral pathogen each year in the United States, with over 5 million of those cases attributed to contaminated foods (Hall *et al*. 2013, 2014). Foods can become contaminated with HuNoV by direct contact with faecal matter on the hands of food handlers. Contaminated hands can also facilitate transfer of virus via high touch and food preparation surfaces (Rönnqvist *et al*. 2014; Grove *et al*. 2015). Therefore, effective hand hygiene is essential in interrupting HuNoV transmission. The main barrier to study the efficacy of treatments to control and inactivate HuNoV has been the lack of an *in vitro* cultivation cell culture system, despite almost 50 years of research attempting to develop such a model. Instead, surrogates such as murine norovirus, feline calicivirus, virus‐like particles (VLPs) or bacterial phages (MS2) have been used (Macinga *et al*. 2008; Park *et al*. 2010; Cromeans *et al*. 2014; Hoelzer *et al*. 2016; Tung *et al*. 2016). Preceding molecular amplification with an RNase pre‐treatment (RNase‐RT‐qPCR) has been used in an effort to better distinguish infectious from non‐infectious RNA virus particles after an inactivation treatment (Tung *et al*. 2016; Manuel *et al*. 2017; Montazeri *et al*. 2017). In this case, the free RNA from compromised viral capsids is degraded by RNase and theoretically, only the intact virus particles (infectious) are quantified.

Successful HuNoV cultivation in stem cell‐derived human intestinal enteroids (HIE) was recently reported (Ettayebi *et al*. 2016). Its applicability to evaluating disinfection efficacy is of interest but is complicated by cell cytotoxicity, limited assay sensitivity, lack of the assay’s ability to quantify log_10_ reductions, reagent availability and cost. The purpose of this study was to evaluate the efficacy of two commercial alcohol‐based hand sanitizers and a 60% ethanol (v/v) control benchmark in inactivating HuNoV using the RNase‐RT‐qPCR method, with subsequent validation of product efficacy using the HIE model.

## Results and discussion

### *In vitro* suspension assay followed by RNase/RT‐qPCR to test hand sanitizer efficacy

The commercial products tested by suspension assay followed by RNase‐RT‐qPCR were more efficacious against HuNoV (sample NV14‐017) compared to the benchmark of 60% ethanol (Table 1; *P* < 0·05). In addition, when there was no additional soil added to the suspension (~2·5% faecally derived residual soil only), Product A outperformed Product B (*P* < 0·05). These results are consistent with those of another study (Park *et al*. 2010), which found that exposure to 70% ethanol produced variable reduction in viral RNA, ranging from 0·5 to 3·0 log_10_ GEC reduction for GII.4 HuNoV strains. A prior study characterizing the efficacy of various concentrations of ethanol and an alcohol‐based hand sanitizer on HuNoV in suspension and on human fingerpads respectively also using the RNase‐RT‐qPCR method, showed limited product efficacy (Liu *et al*. 2009). In that study, <0·5 log_10_ reduction in HuNoV GEC was shown for ethanol suspensions, and a <0·4 log_10_ reduction in HuNoV GEC was shown for fingerpad studies with the commercial hand sanitizer tested. The efficacy of alcohol‐based hand sanitizers against HuNoV has been debated in the literature, and in the absence of an *in vitro* assay to determine infectivity, researchers have had to rely on RNase‐RT‐qPCR or cultivable surrogates to estimate product efficacy (Macinga *et al*. 2008; Liu *et al*. 2009; Park *et al*. 2010; Shimizu‐Onda *et al*. 2013; Cromeans *et al*. 2014; Tuladhar *et al*. 2015).

**Table 1:** Efficacy of commercial hand sanitizers and 60% ethanol against human norovirus in suspension, with and without added soil, as evaluated by RNase/RT‐qPCR

Soil load (%)	Contact time (s)	Product	Log_10_ reduction in HuNoV GEC
2·5	30	Product A	2·9 ± 0·2^a^
		Product B	2·6 ± 0·1^b^
		60% Ethanol	0·9 ± 0·1^c^
2·5	60	Product A	3·2 ± 0·09^a^
		Product B	2·6 ± 0·4^b^
		60% Ethanol	0·9 ± 0·05^c^
5·0	30	Product A	2·3 ± 0·1^a^
		Product B	2·2 ± 0·07^a^
		60% Ethanol	0·7 ± 0·1^b^
5·0	60	Product A	2·6 ± 0·07^a^
		Product B	2·4 ± 0·2^a^
		60% Ethanol	0·6 ± 0·02^b^

Different superscript letters indicate significant differences in results between products for a single treatment type (*P* < 0·05).

### *In vitro* suspension assay followed by HIE challenge to test hand sanitizer efficacy

Optimization experiments were necessary before it was possible to use the HIE model to test sanitizer efficacy. In a head‐to‐head comparison, commercial media out‐performed in‐house media, providing higher cell density (80–100 enteroids/field *vs* 20–30 enteroids/field respectively) and almost twofold faster cell growth (7 days *vs* 10–12 days). Although more expensive than the reagents produced in‐house, the commercial media had a streamlining advantage since there was not need to produce our own growth factors, resulting in significant savings in both time and labour costs.

**Figure 1:**
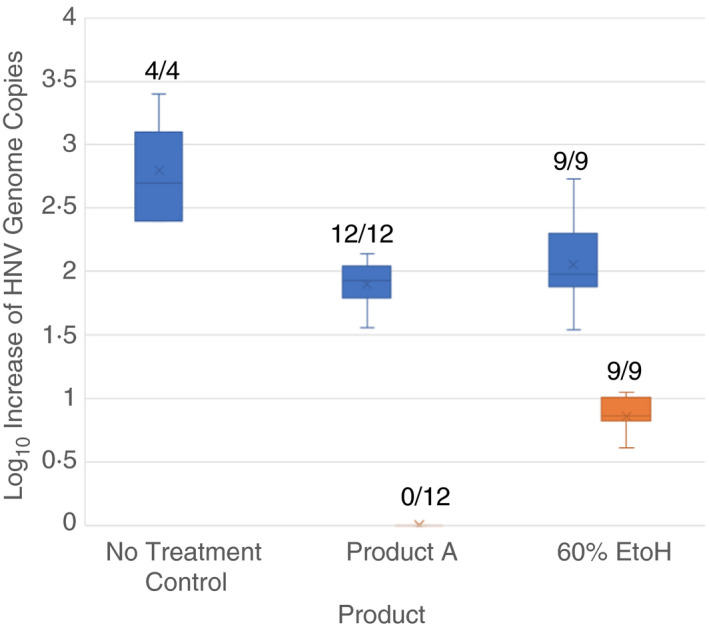
Inactivation of HuNoV by product A and 60% ethanol after a 60 s contact time in suspension assay (ASTM method E1052‐11), tested in HIE model. Each product test was also accompanied by a neutralization control (NC). A no treatment control was also run independently to ensure consistent growth of the HuNoV strain. X denotes the mean, the middle line of the box denotes the median, the top line of the box denotes the 75th percentile, the bottom line of the box denotes the 25th percentile, and whiskers indicate upper and lower values. Fractions listed above the box and whisker plots show number of replicates that showed an increase in viral RNA (evidence of viral replication)/total number of replicates (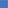 neutralized control; 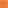 treatment).

Of the two GII.4 Sydney faecal suspensions used in this study, only the NV14‐117 inoculum produced efficient and consistent replication (2·8 ± 0·5 log_10_ GEC increase at 72 hpi; no treatment control–Fig. 1) in the HIE, and only if first purified by serial filtration. The lack of replication of the NV14‐017 inoculum used in the RNase‐RT‐qPCR studies above was not due to differences in the viral RNA polymerase sequence (data not shown). The variable replication efficiency of various HuNoV strains to replicate in HIE has been reported by others (Ettayebi *et al*. 2016; Costantini *et al*. 2018) and appears to be the function of many different factors which range from differences in genotype to faecal sample to faecal sample variation.

Consistently, we found that it was necessary to use some means by which to remove residual disinfectant, surfactants, and/or neutralization buffer prior to challenging the HIE model. Otherwise, extensive cytotoxicity was observed. Treatment with spin columns, as per ASTM E1482‐12, produced a small (0·3 ± 0·15 log_10_ GEC) but insignificant loss in virus titre (*P* > 0·05) compared to the positive control. Even after the use of spin columns for detergent removal, we could not overcome the cytotoxic effect of Product B in the HIE model, so further confirmation of the efficacy of this product by infectivity assay was not possible. This highlights the sensitivity of the HIE model to residual cytotoxic substances and the need to investigate alternative ways to more effectively remove myriad residual formulation components to facilitate more universal use of the HIE model in future disinfection studies.

In the HIE model, neutralization controls produced a 1·97 ± 0·27 log_10_ HuNoV GEC increase (Fig. 1), suggesting the assay provided about 2 log_10_ replication efficiency, which is similar to that reported in the literature (Ettayebi *et al*. 2016; Costantini *et al*. 2018). No virus replication in HIE was observed after treating GII.4 Sydney NV14‐117 with Product A as evidenced in the absence of a RT‐qPCR signal in the 72‐h post infection (hpi) assays (Fig. 1). At 1 hpi, 1·72 ± 0·50 log_10_ HuNoV GEC was detected from samples treated with Product A, in comparison to 3·60 ± 0·60 and 3·41 ± 0·21 log_10_ HuNoV GEC RT‐qPCR signal from the positive control samples and 60% ethanol samples respectively (data not shown), suggesting that treatment with Product A affected capsid binding efficiency. The samples treated with 60% ethanol produced an RT‐qPCR signal consistent with a 0·86 ± 0·16 log_10_ GEC increase at 72 hpi, showing that ethanol produced some inactivation of HuNoV but did not completely eliminate its infectivity (all six of six replicates showing viral replication). This is consistent with another study which showed slightly reduced HuNoV inactivation (up to 0·7 log_10_ GEC lower (72 hpi) than the neutralization control) after treatment with ethanol and no inactivation of HuNoV after treatment by isopropanol using the HIE model (Costantini *et al*. 2018).

It should be noted that the virus inactivation data collected using RNase‐RT‐qPCR and the HIE model are not directly comparable, and the use of the HIE model in this study was to validate the RNase‐RT‐qPCR results. While the HuNoV GII.4 Sydney genotype was used in both studies, the inocula consisted of two unique faecal suspensions obtained from two different infected individuals. Preliminary screening of several GII.4 Sydney‐positive stool samples revealed that sample ID NV14‐117 showed superior HIE replication efficiency, but for unknown reasons. Others have reported similar sample‐to‐sample variation in performance on the HIE model, even when the HuNoV strains were the same genotype (Ettayebi *et al*. 2016; Costantini *et al*. 2018). For the HIE model, it was also necessary to purify NV14‐117 by serial filtration prior to exposure to the sanitizers, consistent with other studies (Ettayebi *et al*. 2016). This step likely resulted in removal of some residual organic matter associated with the resuspended stool, as well as disaggregation of viral particles (Escudero‐Abarca *et al*. 2014). This could have provided conditions more favourable to virus inactivation compared to the unfiltered 20% faecal suspension used in the RNase‐RT‐qPCR experiments. Unfortunately, these sample manipulations were necessary in order to effectively use the HIE model for this application.

One of the major limitations of the current HIE model for HuNoV is its lack of quantifiability. Previous studies investigating the efficacy of alcohol‐based hand sanitizers against HuNoV surrogates report results as log_10_ reductions thanks to the availability of quantifiable infectivity assays, such as plaque and TCID_50_ assays (Macinga *et al*. 2008; Park *et al*. 2010; Shimizu‐Onda *et al*. 2013). In order to develop a quantifiable infectivity assay for HuNoV disinfection studies, a greater understanding of the mechanism of infection of HuNoV in the intestinal epithelium is required. The cellular factors that regulate HuNoV binding and uptake into intestinal cells for successful infection are still unknown (Haga *et al*. 2020). Ongoing research in this field is needed to develop a quantifiable infectivity assay for HuNoV.

## Conclusions

It is possible to use the current HIE model to screen the efficacy of sanitizers and disinfectants against HuNoV in a qualitative fashion. These data can provide evidence supporting product efficacy as determined quantitatively using RNase‐RT‐qPCR assays. Due to the cost of commercial media, time required to run the assays, and its lack of quantifiability, the ‘every day’ use of the HIE model for the broad screening of product efficacy is impractical. The use of RNase‐RT‐qPCR based assays for broad screening of product efficacy against HuNoV in a quantitative fashion is still of value, until such time as the HIE cultivation model is simplified and progresses to providing log_10_ reductions using plaque assays or TCID_50_ assays. The hand sanitizers evaluated in this study were found to be significantly more efficacious in inactivating HuNoV compared to the 60% ethanol benchmark in suspension assay (*P* < 0·05), and these results could be validated by the HIE model. The ability to effectively validate molecular data using an infectivity model is an important advancement toward more reliable characterization of anti‐HuNoV efficacy of sanitizers and disinfectants. This work further demonstrates that formulation is an important consideration in production of alcohol‐based hand sanitizers with efficacy against HuNoV.

## Materials and methods

### HuNoV strains

GII.4 Sydney strains obtained as deidentified stool specimens from outbreaks were suspended 20% in PBS with clarification by centrifugation (3100 ***g*** for 5 min at 4°C). Residual soil (organic) load in the working suspensions was approximately 2·5% as previously reported (Moorman *et al*. 2017). Samples obtained from two different naturally infected individuals were used in this study, both with initial titres of 6–7 log_10_ GEC per ml. Sample designation NV14‐017 was used without further preparation for *in vitro* suspension assays evaluated by RNase‐RT‐qPCR. Sample designation NV14‐117 was used in suspension assays tested using the HIE model. To facilitate infection and reduce cytotoxicity in the HIE studies, it was necessary to further purify this stock by serial filtration through 0·8, 0·45 and 0·22 µm filters (Millipore Sigma, Burlington, MA) prior to treatment with disinfectants, consistent with previous studies (Ettayebi *et al*. 2016).

### Products screened

Two commercial alcohol‐based hand sanitizers (GOJO Industries, Inc., Akron, OH), PURELL^®^ VF PLUS™ (Product A) and PURELL VF 481™ (Product B) and 60% ethanol (v/v) were screened in parallel in this study (Table 2) by *in vitro* suspension assay (ASTM E1052‐11) followed by evaluation of virus inactivation (i) directly by RNase‐RT‐qPCR; and (ii) by HIE culture with pre‐ and post‐infection RT‐qPCR.

**Table 2:** Products screened in this study and their ingredients

Test product	Active ingredient	Inert ingredients	Product format
Product A	85% Ethanol	Water, isopropanol, isopropyl myristate, caprylyl glycol, aminomethyl propanol, acrylates/C10‐30, alkyl acrylate crosspolymer	Gel
Product B	70% Ethanol	Water, isopropanol, copper gluconate, diisopropyl sebacate, PEG/PPG‐20/6 dimethicone, pentaerythrityl tetra‐di‐t‐butyl hydroxyhydrocinnamate, polyquaternium‐37	Gel
60 % Ethanol (Benchmark)	60% Ethanol		Liquid

### *In vitro* suspension assay followed by RNase‐RT‐qPCR for evaluation of product efficacy

Virucidal suspension assays of GII.4 Sydney sample NV14‐017 for the *in vitro* study were done in accordance with ASTM standard E1052‐11, with minor modifications. Briefly, a 25 µl volume of the 20% virus faecal suspension, and the same volume of the faecal suspension supplemented with an additional 2·5% soil (prepared according to ASTM standard E1053‐11, for an approximate total soil load of 5%) was mixed with 225 µl of the products, 60% ethanol, or PBS. After 30 and 60 s contact times, 20 µl of this solution was added to 180 µl of 10% D/E neutralization broth (Sigma‐Aldrich, St Louis, MO). Suspensions were held frozen at −80°C until assayed. Prior to RNA extraction, samples were treated with RNase to eliminate free RNA. For RNase pre‐treatment, 2 μl RNase One (Promega, Madison, WI) and 22 μl reaction buffer were added to 200 μl of the post‐neutralization sample eluate and incubated at 37°C for 15 min. Samples were placed on ice for 5 min to abolish RNase enzyme activity prior to RNA extraction, which was performed using the automated NucliSENS^®^ easyMag^®^ system (bioMerieux, Durham, NC) as per the manufacturer’s instructions. RNA was eluted in a 25 μl volume of proprietary NucliSENS elution buffer. Viral RNA was quantified by RT‐qPCR targeting the conserved OFR1‐ORF2 junction of GII HuNoV (Jothikumar *et al*. 2005). The resulting C_T_ values were extrapolated to log_10_ genome equivalent copies (GEC) by comparison to a standard curve produced by RT‐qPCR amplification of HuNoV GII.4 Sydney RNA obtained from the initial inoculum. Results were presented as log_10_ reduction in GEC of HuNoV.

### Human intestinal enteroid monolayer production

Human intestinal enteroids (HIE; jejunal) were kindly provided by Dr Mary Estes, Baylor College of Medicine (BCM), Houston, TX. They were initially grown as multilobular 3D cultures in matrigel using in‐house media and were passaged after 7 days. In‐house media required maintenance of three separate cell lines to produce key growth factors: (i) Wnt3A cells (ATCC, CRL‐2647); (ii) 293T‐HA Rpo‐FC cells to produce R‐Spondin (Trevigen, Gaithersburg, MD); and (iii) 293 Noggin cells (provided by BCM colleagues). Complete media with growth factors (CMGF+) was required to grow undifferentiated enteroids, and CMGF+ with omission/reduction of key growth factors (CMGF−) was required to obtain differentiated enteroids. This system (Ettayebi *et al*. 2016) was simplified with the recent availability of commercial media (Intesticult, STEMCELL, Technologies, Kent, WA) used as per manufacturer recommendations.

Three‐dimensional cultures were dissociated into single cell suspensions in CMGF+ supplemented with 10 µmol l^−1^ Y‐27632 (Sigma Aldrich, St Louis, MO) and plated into 96‐well plates coated with collagen IV (Corning Life Sciences, Tewksbury, MA) at a seeding density of 1–2 × 10^5^ cells/well as undifferentiated monolayers (Costantini *et al*. 2018). After 48 h the media was replaced with differentiation medium and after 4 days of incubation, a confluent monolayer of differentiated enteroids was available for infection studies.

### *In vitro* suspension assay followed by HIE culture for evaluation of product efficacy

The virucidal suspension assays performed in this phase of the study were similar to the ASTM E1052‐11 described above except for: (i) the use of highly purified HuNoV GII.4 Sydney sample NV14‐117, which showed superior replication efficiency in the HIE model (data not shown); (ii) a smaller inoculum volume (10 µl) was used, with associated volume reduction for sanitizer and neutralization; (iii) CMGF‐ supplemented with 10% faetal bovine serum was used as the neutralizer; (iv) only Product A and 60% ethanol at the 60 s contact time without soil was tested; and (v) after neutralization, the samples were purified using detergent removal spin columns (Thermofisher, Rockford, IL) to reduce residual cytotoxicity, as per ASTM E1482‐12. Product B produced cytotoxic effects in the model despite the use of the detergent removal spin columns, so further studies with this product in the HIE model were not possible.

### HIE infection

The positive control consisted of purified HuNoV GII.4 Sydney, strain NV14‐117, having a titre of 6–7 log_10_ GEC per ml. Monolayer cultures were infected with 100 µl of diluted (i) untreated virus stock (no treatment control); (ii) virus stock exposed to neutralized product (neutralization control); and (iii) virus stock exposed to product followed by neutralization (treatment). These were then incubated for 1 h to facilitate virus binding, followed by washing with CMGF‐ and overlay with 100 µl of differentiation media containing 500 µmol l^−1^ sodium glycochenodeoxycholate (Sigma Aldrich). For each set of infections, duplicate plates were prepared; one plate was removed and stored at −80°C immediately post infection (corresponding to 1 h post‐infection (hpi)) and another was incubated for 72 h prior to freezing (constituting 72 hpi). Frozen plates were subjected to RNA extraction using the Direct‐zol RNA kit (Zymo Research, Irvine, CA) according to the manufacturer’s instructions, with RNA elution in a 25 µl volume of DNase/RNase free water. Viral RNA was quantified by RT‐qPCR targeting the conserved ORF1‐ORF2 junction of GII HuNoV (Jothikumar *et al*. 2005). The resulting C_T_ values were extrapolated to log_10_ GEC by comparison to a standard curve produced by RT‐qPCR amplification of HuNoV GII.4 Sydney RNA obtained from the initial inoculum. Results were presented as log_10_ increase in GEC of HuNoV.

### Statistical analysis

Suspension assay experiments with RNase‐RT‐qPCR enumeration were done in independent triplicates. Suspension assays followed by HIE infectivity assays were completed in two wells for two to six independent runs, yielding 4–12 replicates. When RNase‐RT‐qPCR was used as the quantification method, results were expressed as mean ± standard deviation of log_10_ reduction in HuNoV GEC. When the HIE model was used, log_10_ HuNoV GEC increase after 72 hpi was used to assess whether HuNoV infectivity was abolished by sanitizer treatment. Results were compared statistically using anova and the Tukey‐Kramer test (Sigma Plot; Systat Software Inc., San Jose, CA). Statistical significance was established at a level of *P* < 0·05.

## References

[lam13393-bib-0001] Costantini, V. , Morantz, E.K. , Browne, H. , Ettayebi, K. , Zeng, X.‐L. , Atmar, R.L. , Estes, M.K. and Vinjé, J. (2018) Human norovirus replication in human intestinal enteroids as model to evaluate virus inactivation. Emerg Infect Dis 24, 1453–1464. 30014841 10.3201/eid2408.180126 PMC6056096

[lam13393-bib-0002] Cromeans, T. , Park, G.W. , Costantini, V. , Lee, D. , Wang, Q. , Farkas, T. , Lee, A. and Vinjé, J. (2014) Comprehensive comparison of cultivable norovirus surrogates in response to different inactivation and disinfection treatments. Appl Environ Microb 80, 5743–5751. 10.1128/AEM.01532-14 PMC4178592 25015883

[lam13393-bib-0003] Escudero‐Abarca, B.I. , Rawsthorne, H. , Goulter, R.M. , Suh, S.H. and Jaykus, L.A. (2014) Molecular methods used to estimate thermal inactivation of a prototype human norovirus: more heat resistant than previously believed? Food Microbiol 41, 91–95. 24750817 10.1016/j.fm.2014.01.009

[lam13393-bib-0004] Ettayebi, K. , Crawford, S.E. , Murakami, K. , Broughman, J.R. , Karandikar, U. , Tenge, V.R. , Neill, F.H. , Blutt, S.E. *et al* (2016) Replication of human noroviruses in stem cell–derived human enteroids. Science 353, 1387–1393. 27562956 10.1126/science.aaf5211 PMC5305121

[lam13393-bib-0005] Glass, R.I. , Parashar, U.D. and Estes, M.K. (2009) Norovirus gastroenteritis. New Engl J Med 361, 1776–1785. 19864676 10.1056/NEJMra0804575 PMC3880795

[lam13393-bib-0006] Grove, S.F. , Suriyanarayanan, A. , Puli, B. , Zhao, H. , Li, M. , Li, D. , Schaffner, D.W. and Lee, A. (2015) Norovirus cross‐contamination during preparation of fresh produce. Int J Food Microbiol 198, 43–49. 25590260 10.1016/j.ijfoodmicro.2014.12.023

[lam13393-bib-0007] Haga, K. , Ettayebi, K. , Tenge, V.R. , Karandikar, U.C. , Lewis, M.A. , Lin, S.‐C. , Neill, F.H. , Ayyar, B.V. *et al* (2020) Genetic manipulation of human intestinal enteroids demonstrates the necessity of a functional fucosyltransferase 2 gene for secretor‐dependent human norovirus infection. MBio 11, 10.1128/mBio.00251-20. PMC7078471 32184242

[lam13393-bib-0008] Hall, A.J. , Lopman, B.A. , Payne, D.C. , Patel, M.M. , Gastañaduy, P.A. , Vinjé, J. and Parashar, U.D. (2013) Norovirus disease in the United States. Emerg Infect Dis 19, 1198–1205. 23876403 10.3201/eid1908.130465 PMC3739528

[lam13393-bib-0009] Hall, A.J. , Wikswo, M.E. , Pringle, K. , Gould, L.H. and Parashar, U.D. (2014) Vital signs: foodborne norovirus outbreaks — United States, 2009–2012. MMWR 63, 491–495. 24898166 PMC5779359

[lam13393-bib-0010] Hoelzer, K. , Fanaselle, W. , Pouillot, R. , Doren, J.M.V. and Dennis, S. (2016) Virus inactivation on hard surfaces or in suspension by chemical disinfectants: systematic review and meta‐analysis of norovirus surrogates. J Food Protect 76, 1006–1016. 10.4315/0362-028X.JFP-12-438 23726196

[lam13393-bib-0011] Jothikumar, N. , Lowther, J.A. , Henshilwood, K. , Lees, D.N. , Hill, V.R. and Vinjé, J. (2005) Rapid and sensitive detection of noroviruses by using TaqMan‐based one‐step reverse transcription‐PCR assays and application to naturally contaminated shellfish samples. Appl Environ Microb 71, 1870–1875. 10.1128/AEM.71.4.1870-1875.2005 PMC1082570 15812014

[lam13393-bib-0012] Liu, P. , Yuen, Y. , Hsiao, H.‐M. , Jaykus, L.‐A. and Moe, C. (2009) Effectiveness of liquid soap and hand sanitizer against norwalk virus on contaminated hands. Appl Environ Microbiol 76, 394–399. 19933337 10.1128/AEM.01729-09 PMC2805232

[lam13393-bib-0013] Macinga, D.R. , Sattar, S.A. , Jaykus, L.‐A. and Arbogast, J.W. (2008) Improved inactivation of nonenveloped enteric viruses and their surrogates by a novel alcohol‐based hand sanitizer. Appl Environ Microbiol 74, 5047–5052. 18586970 10.1128/AEM.00487-08 PMC2519287

[lam13393-bib-0021] Manuel, C.S. , Moore, M.D. & Jaykus, L. ‐A. (2017) Efficacy of a disinfectant containing silver dihydrogen citrate against GI.6 and GII.4 human norovirus. J Appl Microbio 122, 78–86. 10.1111/jam.13331 27775827

[lam13393-bib-0014] Moorman, E. , Montazeri, N. and Jaykus, L.‐A. (2017) Efficacy of neutral electrolyzed water for inactivation of human norovirus. Appl Environ Microbiol 83, 10.1128/AEM.00653-17. PMC5541222 28600317

[lam13393-bib-0022] Montazeri, N. , Manuel, C. , Moorman, E. , Khatiwada, J.R. , Williams, L.L. & Jaykus, L.‐A. (2017) Virucidal Activity of Fogged Chlorine Dioxide‐ and Hydrogen Peroxide‐Based Disinfectants against Human Norovirus and Its Surrogate, Feline Calicivirus, on Hard‐to‐Reach Surfaces. Front Microbiol 8, 1031 28642746 10.3389/fmicb.2017.01031 PMC5462988

[lam13393-bib-0015] Park, G.W. , Barclay, L. , Macinga, D. , Charbonneau, D. , Pettigrew, C.A. and Vinjé, J. (2010) Comparative efficacy of seven hand sanitizers against murine norovirus, feline calicivirus, and gii.4 norovirus. J Food Protect 73, 2232–2238. 10.4315/0362-028x-73.12.2232 21219741

[lam13393-bib-0016] Rönnqvist, M. , Aho, E. , Mikkelä, A. , Ranta, J. , Tuominen, P. , Rättö, M. and Maunula, L. (2014) Norovirus transmission between hands, gloves, utensils, and fresh produce during simulated food handling. Appl Environ Microbiol 80, 5403–5410. 24951789 10.1128/AEM.01162-14 PMC4136105

[lam13393-bib-0017] Scallan, E. , Hoekstra, R.M. , Angulo, F.J. , Tauxe, R.V. , Widdowson, M.‐A. , Roy, S.L. , Jones, J.L. and Griffin, P.M. (2011) Foodborne illness acquired in the United States—major pathogens. Emerg Infect Dis 17, 7–15. 21192848 10.3201/eid1701.P11101 PMC3375761

[lam13393-bib-0018] Shimizu‐Onda, Y. , Akasaka, T. , Yagyu, F. , Komine‐Aizawa, S. , Tohya, Y. , Hayakawa, S. and Ushijima, H. (2013) The virucidal effect against murine norovirus and feline calicivirus as surrogates for human norovirus by ethanol‐based sanitizers. J Infect Chemother 19, 779–781. 23135829 10.1007/s10156-012-0516-2

[lam13393-bib-0019] Tuladhar, E. , Hazeleger, W.C. , Koopmans, M. , Zwietering, M.H. , Duizer, E. and Beumer, R.R. (2015) Reducing viral contamination from finger pads: handwashing is more effective than alcohol‐based hand disinfectants. J Hosp Infect 90, 226–234. 25936671 10.1016/j.jhin.2015.02.019

[lam13393-bib-0020] Tung, G. , Macinga, D. , Arbogast, J. and Jaykus, L.‐A. (2016) Efficacy of commonly used disinfectants for inactivation of human noroviruses and their surrogates. J Food Protect 76, 1210–1217. 10.4315/0362-028X.JFP-12-532 23834796

